# Recent and Forecasted Increases in Coccidioidomycosis Incidence Linked to Hydroclimatic Swings, California, USA

**DOI:** 10.3201/eid3105.241338

**Published:** 2025-05

**Authors:** Simon K. Camponuri, Alexandra K. Heaney, Gail Sondermeyer Cooksey, Duc J. Vugia, Seema Jain, Daniel L. Swain, John Balmes, Justin V. Remais, Jennifer R. Head

**Affiliations:** University of California, Berkeley, California, USA (S.K. Camponuri, J. Balmes, J.V. Remais); University of California, San Diego, California, USA (A. K. Heaney); California Department of Public Health, Richmond, California, USA (G.S. Cooksey, D.J. Vugia); San Francisco Department of Public Health, San Francisco, California, USA (S. Jain); University of California Agriculture and Natural Resources, Davis, California, USA (D.L. Swain); University of California, Los Angeles, California, USA (D.L. Swain); NSF National Center for Atmospheric Research, Boulder, Colorado, USA (D.L. Swain); University of California, San Francisco (J. Balmes); University of Michigan, Ann Arbor, Michigan, USA (J.R. Head)

**Keywords:** coccidioidomycosis, fungi, Valley fever, forecast, infectious disease, California, ensemble modeling, hydroclimate, precipitation variability, climate change, United States

## Abstract

In 2023, California reported near–record high coccidioidomycosis cases after a dramatic transition from drought to heavy precipitation. Using an ensemble model, we forecasted 12,244 cases statewide during April 1, 2024–March 31, 2025, a 62% increase over cases reported 2 years before and on par with case counts for 2023.

Incidence of coccidioidomycosis, an emerging infectious disease caused by *Coccidioides* spp. fungi, has increased dramatically since 2000 (*1*). In 2023, California, USA, reported a near-record 9,054 coccidioidomycosis cases (only surpassed by 9,093 cases in 2019). During April 2023–March 2024, a period capturing the full seasonal rise and fall in incidence, California reported 10,519 cases, 39% higher than the same period the previous year (*2*).

The 2023 spike in incidence might be attributable, in part, to a swing from extreme drought to heavy precipitation during winter 2022–2023. Transitions from dry to wet years have been linked to increased coccidioidomycosis incidence (*3*,*4*). Soil moisture during wet winters is hypothesized to support fungal growth, contributing to an abundance of spores available for airborne dispersal during hot, dry conditions characteristic of summer and early fall (*3*). Drought preceding rainy seasons might enhance fungal growth by eliminating microbial competitors from soils (*5*) or by affecting rodent populations, a putative reservoir host and nutrient source for the fungus (*6*). During 2020–2022, California experienced severe drought (*7*). An unusually wet winter followed in 2022–2023; statewide precipitation exceeded 150% of average, among the top 10 wettest seasons in the past century (*7*). Statewide precipitation during the 2023–2024 wet season was 115% of the long-term average, marking the second consecutive wetter-than-average season after a severe drought (*7*). That pattern suggested high coccidioidomycosis incidence might continue throughout the 2024 transmission year. We developed a disease forecast to guide public health alerts and messages by pinpointing when and where disease risk is expected to be highest. Such targeted messaging can raise awareness about disease risk, leading to earlier diagnosis and more effective disease management (*8*).

## The Study

We adapted our previously published ensemble prediction model relating monthly reported cases per census tract to climatologic or environmental predictors (Appendix Table 1) (*3*). Using a progressive time-series cross validation approach (Appendix Figure 1), we examined how each of 5 candidate algorithms performed when forecasting future out-of-sample cases and calculated an ensemble weight for each candidate model as proportional to the inverse of each model’s mean out-of-sample prediction error (Appendix Table 2) (*9*). We fit separate models for each county (for highly endemic regions) or region (for low to moderately endemic regions) to account for spatial differences in the effects of precipitation and temperature on coccidioidomycosis incidence (Appendix Figure 2). Within random forest models, temperature had high variable importance in forecasting cases in wetter, coastal regions, whereas precipitation had high importance in the drier Central Valley (Appendix Figure 3). Those results align with previous findings and emphasize the role of wet periods for fungal growth and hot, dry periods for spore dispersal (*3*).

To forecast coccidioidomycosis cases, we applied each model to temperature and precipitation data from January 2023–March 2024 and extrapolated temperature and precipitation data through March 2025 (*10*). We generated future temperature and precipitation estimates beyond April 2024 (the date of analysis) by extrapolating historical monthly temperature averages in each census tract using a 42-year linear trend (1981–2023) and by setting precipitation to the 50th percentile of the 42-year precipitation distribution (*10*). Because future climate is unknown, we examined the sensitivity of forecasts to 2 alternative precipitation scenarios, drier-than-average (20th percentile) and wetter-than-average (80th percentile), and 2 temperature scenarios, warmer-than-average (+3°F) and cooler-than-average (−3°F). We fit each model to data from January 1, 2000–December 31, 2022, and created an ensemble forecast of cases for each month during January 1, 2023–March 31, 2025, by taking the weighted average of each model’s forecast. We summed cases across calendar years and the coccidioidomycosis transmission year (*2*), which spans April 1 of 1 year to March 31 of the next. To quantify uncertainty in our forecasts, we generated 90% prediction intervals (PIs) using a 2-step bootstrapping process (Appendix Figure 4). All analyses were conducted in R v.4.3.2 (The R Project for Statistical Computing, https://www.r-project.org).

**Figure 1:**
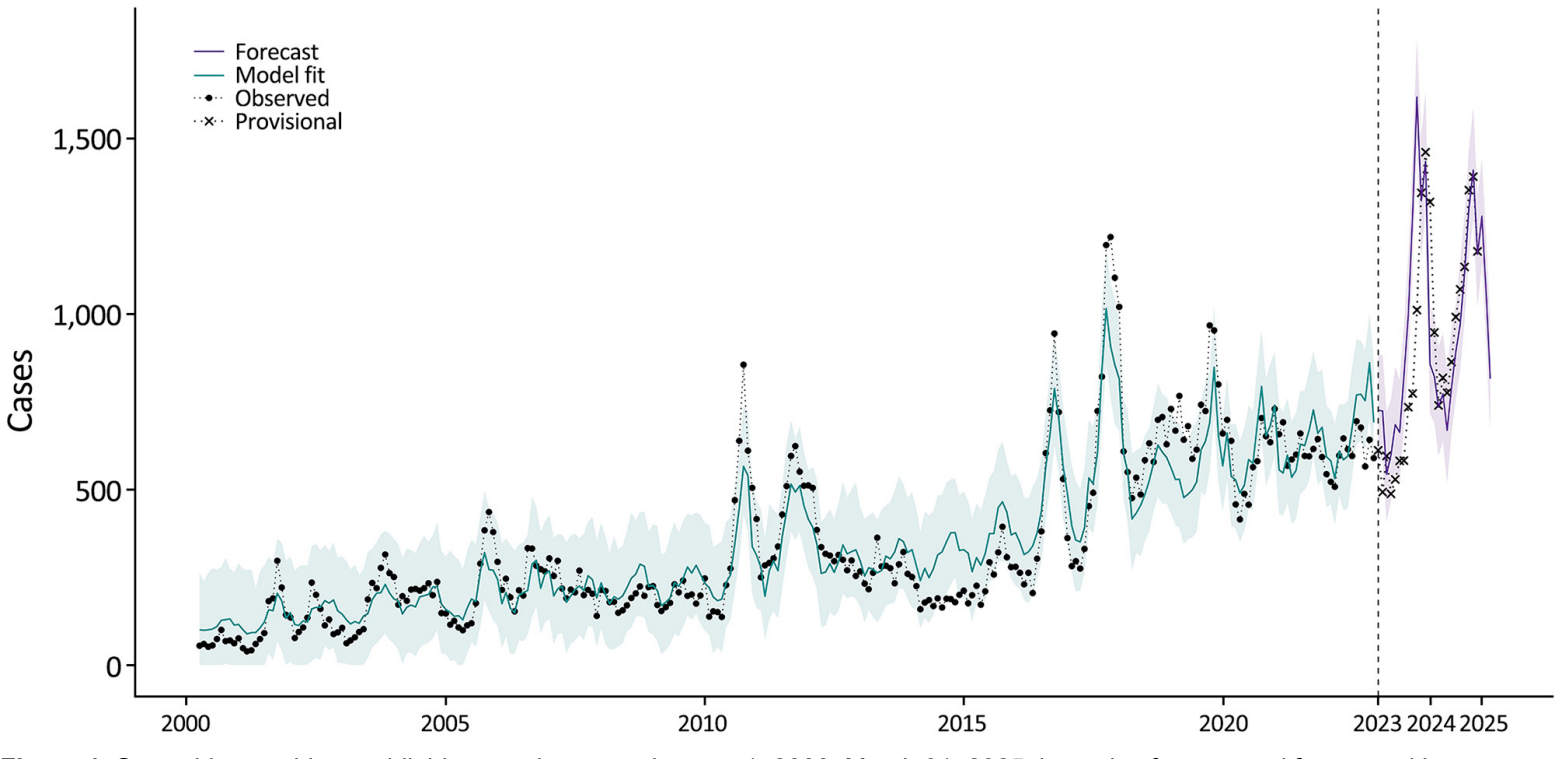
Statewide monthly coccidioidomycosis cases, January 1, 2000–March 31, 2025, in study of recent and forecasted increases in coccidioidomycosis incidence linked to hydroclimatic swings, California, USA. Black dots indicate confirmed cases reported during 2000–2022, times symbols indicate the provisional cases reported during January 1, 2023–December 31, 2024, the green line represents the ensemble model fit to the observed case data (R^2^ = 0.87), and the purple line indicates the ensemble model predicted (April 1, 2023–March 31, 2024) and forecasted (April 1, 2024–March 31, 2025) cases. Shading represents 90% prediction intervals.

**Figure 2:**
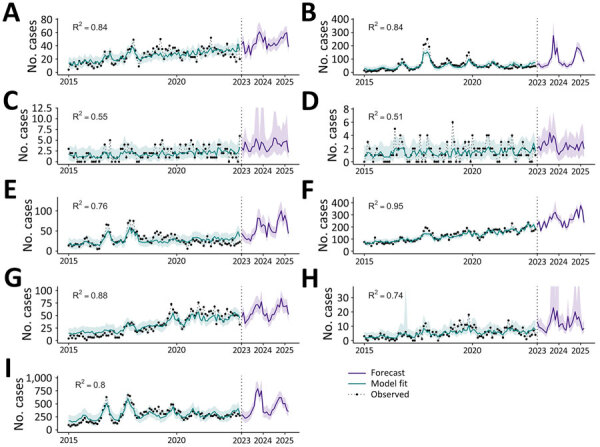
Regional monthly coccidioidomycosis cases, January 1, 2015–March 31, 2025, in study of recent and forecasted increases in coccidioidomycosis incidence linked to hydroclimatic swings, California, USA. A) Bay Area; B) Central Coast; C) Eastern California; D) Northern California; E) Northern San Joaquin Valley; F) Southern Coast; G) Southern Inland; H) Southern Sacramento Valley; I) Southern San Joaquin Valley. Black dots indicate confirmed cases reported during 2015–2022, green line represents the ensemble model fit to the observed case data, and the purple line indicates the ensemble model predicted (April 1, 2023–March 31, 2024) and forecasted (April 1, 2024–March 31, 2025) cases during April 1, 2023–March 31, 2025. Shading represents 90% prediction intervals.

Our ensemble forecast model predicted 11,846 cases (90% PI 11,261–12,505) in California during April 1, 2023–March 31, 2024, closely matching the preliminary state report of 10,519 (Figure 1) (*2*). The reported number of monthly cases in California in 2023 peaked at 1,462, aligning with our model forecast of 1,619 (90% PI 1,410–1,745). Our forecast model predicted the highest case counts in the Southern San Joaquin Valley, Southern Coast, and Central Coast (Table, Figure 2). Although our model slightly overpredicted cases in these regions, our predictions aligned with the relative magnitude of cases across regions (Appendix Table 3).

**Table:** Region-level forecasted incident cases for the 2023 (April 2023–March 2024) and 2024 (April 2024–March 2025) transmission years in study of recent and forecasted increases in coccidioidomycosis incidence linked to hydroclimatic swings, California, USA*

Region	2023 forecasted (90% PI)	2024 forecasted (90% PI)
Bay Area	529 (482–589)	558 (512–610)
Central Coast	1,189 (1,025–1,400)	1,207 (1,071–1,378)
Eastern California	42 (30–76)	49 (32–82)
Northern California	32 (22–43)	28 (19–38)
Northern San Joaquin Valley	605 (515–703)	811 (696–924)
Southern Coast	3,049 (2,875–3,269)	3,322 (3,172–3,494)
Southern Inland	694 (625–770)	725 (658–797)
Southern San Joaquin Valley	5,557 (5,084–6,182)	5,399 (4,993–5,902)
Southern Sacramento Valley	149 (120–542)	147 (120–255)
Statewide	11,846 (11,224–12,456)	12,244 (11,638–12,917)

*PI, prediction interval.

**Figure 3:**
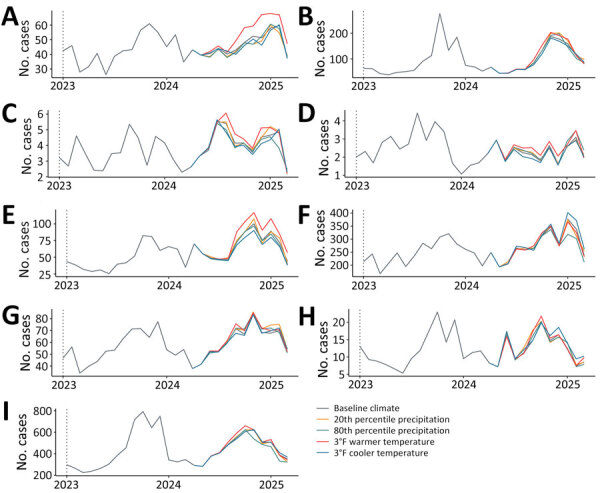
Forecasted regional monthly coccidioidomycosis cases during January 1, 2023–March 31, 2025, under varying future climates in 2024–2025 in study of recent and forecasted increases in coccidioidomycosis incidence linked to hydroclimatic swings, California, USA. A) Bay Area; B) Central Coast; C) Eastern California; D) Northern California; E) Northern San Joaquin Valley; F) Southern Coast; G) Southern Inland; H) Southern Sacramento Valley; I) Southern San Joaquin Valley. The baseline climate scenario represents the 50th percentile of precipitation during 1981–2023 and extrapolated monthly average temperatures assuming a 42-year linear trend. The 20th and 80th percentile precipitation scenarios assume the baseline temperature scenario, and the 3°F warmer or cooler temperature scenarios assume the baseline precipitation scenario (i.e., 50th percentile).

Our model forecasted 12,244 cases (90% PI 11,579–12,964) statewide during April 1, 2024–March 31, 2025, a 62% increase over the transmission year 2 years before. The Southern San Joaquin Valley (5,399 [90% PI 4,993–5,902] cases), Southern Coast (3,322 [90% PI 3,172–3,494] cases), and Central Coast (1,207 [90% PI 1,071–1,378] cases) were expected to have the largest number of infections (Table; Figure 2). Our model forecasted pronounced seasonality in disease incidence (Figure 2), with incidence beginning to rise in June and peaking in November at 1,411 (90% PI 1,267–1,587) cases statewide, 98% higher than the 2022 peak (714) and nearly as high as the 2023 peak (1,462). Forecasts were similar under alternative climate scenarios in the forecasted period (Figure 3), suggesting that previous climate conditions are a larger driver of incidence than concurrent climate. Forecasts were robust to model specifications that modeled year as a natural spline and removed collinear predictors (Appendix Table 4).

## Conclusions

Predictive models that forecast disease risk can provide public health officials and healthcare providers with information about timing, location, and magnitude of future disease risk (*8*). Our forecast of high incidence after a swing from extreme drought to heavy precipitation aligns with previous work showing an association between coccidioidomycosis incidence and transitions from anomalously dry to wet years (*3*). Climate change is altering the hydroclimate of California and adjacent regions, with implications for coccidioidomycosis (*11*,*12*). Although changes in average precipitation in California are likely to be modest (*13*), precipitation variability will likely increase considerably (*14*), including increasingly large and frequent swings between high and low precipitation conditions from season to season and between years, a phenomenon known as precipitation whiplash (*12*).

Risk for *Coccidioides* exposure is likely to be highest in the dry summer and fall months; drier soil leads to more dust and the release of *Coccidioides* spores. Reported cases typically lag pathogen exposure by 1–2 months, aligning with the observed peak of cases around November (*15*). However, risk for infection persists year-round. To prevent exposure, persons in regions where coccidioidomycosis is endemic and emerging should avoid dust where possible, practice dust suppression, and consider using N95 masks when disturbing soil. Clinicians should consider coccidioidomycosis when evaluating a patient with respiratory illness who has spent time in an endemic or emerging region, particularly those who are unresponsive to antibiotics, who had exposure to dust or dirt, or whose symptoms last >1–2 weeks.

This analysis is subject to exposure misclassification because case data were aggregated to disease onset, which lags exposure, and census tract of patient residence, which might not represent exposure location. Climate conditions in the forecasted period are uncertain; however, forecasts were similar under the alternate climate scenarios examined. Future work might consider using seasonal climate predictions from large ensemble climate models. Our forecasts cannot account for stochastic point source outbreaks that might lead to anomalously high case counts in certain regions. Continued collaborative work might focus on developing an accurate coccidioidomycosis forecasting system that can be integrated into public health practice in California and other endemic regions.

This article was originally published as a preprint at https://www.medrxiv.org/content/10.1101/2024.08.30.24312858v1.

Appendix Additional information about recent and forecasted increases in coccidioidomycosis incidence linked to hydroclimatic swings, California, USA.
