# Supplementary material for: Recent and Forecasted Increases in Coccidioidomycosis Incidence Linked to Hydroclimatic Swings, California, USA
Source: Emerg Infect Dis. 2025 May;31(5):1028–32. doi: 10.3201/eid3105.241338 (PMC12044236; doi:10.3201/eid3105.241338)
Supplement: Appendix — Additional information about recent and forecasted increases in coccidioidomycosis incidence linked to hydroclimatic swings, California, USA. [file 24-1338-Techapp-s1.pdf]

Article DOI: https://doi.org/10.3201/eid3105.241338

EID cannot ensure accessibility for supplementary materials supplied by authors. Readers who have difficulty accessing supplementary content should contact the authors for assistance.

Recent and Forecasted Increases in Coccidioidomycosis Incidence Linked to Hydroclimatic Swings, California

Appendix

Appendix Table 1. Variables included in the five models included in the ensemble. Green cells were used as main effects and blue cells were used as interactions. Models 1–4 were generalized linear models (GLMs) and model 5 was a random forest (RF).

Variable	GLM 1	GLM 2	GLM 3	GLM 4	RF
Population (as an offset)	Green	Green	Green	Green	Green
Year	Green	Green	Green	Green	
Season (as a factor)	Green	Green	Green	Green, Blue	Green
Percent sand	Green		Green	Green	Green
Impervious surface	Green		Green	Green	Green
Elevation	Green		Green	Green	Green
Total rainfall					
Lag 1 mo	Green		Green		Green
Lag 3 mo	Green, Blue		Green	Green, Blue	Green
Lag 6 mo	Green, Blue		Green	Green, Blue	Green
Lag 9 mo	Green, Blue		Green	Green, Blue	Green
Lag 12 mo	Green, Blue		Green	Green, Blue	Green
Lag 15 mo	Green		Green		Green
Lag 18 mo	Green		Green		Green
Lag 21 mo	Green		Green		Green
Lag 24 mo	Green		Green	Green, Blue	Green
Lag 27 mo	Green		Green		
Lag 30 mo	Green		Green		
Lag 33 mo	Green		Green		
Lag 36 mo	Green		Green	Green, Blue	
Average Temperature					
Lag 1 mo	Green		Green		Green
Lag 3 mo	Green		Green	Green, Blue	Green
Lag 6 mo	Green		Green	Green, Blue	Green
Lag 9 mo	Green		Green	Green, Blue	Green
Lag 12 mo	Green		Green	Green, Blue	Green
Lag 15 mo	Green		Green		Green
Lag 18 mo	Green		Green		Green
Lag 21 mo	Green		Green		Green
Lag 24 mo	Green		Green		Green
Lag 27 mo	Green		Green		
Lag 30 mo	Green		Green		
Lag 33 mo	Green		Green		
Lag 36 mo	Green		Green		
One year post drought (indicator)	Green				Green
Two years post drought (indicator)	Green				Green

Appendix Table 2. Region-specific model weights used to generate the ensemble model. Weights were obtained using the out of sample prediction error (SSE) for each cross-validation fold and for each candidate algorithm. Using the weighted mean of each candidate algorithms' SSE across all folds, with weights proportional to the number of training years in the fold (e.g., folds with more training years had higher weights), model weights were calculated as the normalized inverse of the weighted mean.

Region	Model 1	Model 2	Model 3	Model 4	Model 5
Bay Area	0.207	0.206	0.207	0.207	0.173
Eastern CA	0.202	0.204	0.202	0.203	0.189
Fresno	0.231	0.353	0.121	0.039	0.255
Kern	0.192	0.199	0.195	0.198	0.215
Kings	0.209	0.191	0.176	0.217	0.207
Madera	0.198	0.203	0.199	0.201	0.199
Merced	0.199	0.212	0.204	0.175	0.211
Monterey	0.243	0.229	0.229	0.053	0.247
Northern CA	0.201	0.203	0.202	0.201	0.193
Sacramento Valley	0.19	0.214	0.213	0.212	0.171
San Joaquin	0.210	0.210	0.189	0.185	0.205
San Luis Obispo	0.199	0.185	0.187	0.217	0.211
Santa Barbara	0.198	0.222	0.192	0.173	0.214
Southern Coast	0.210	0.205	0.210	0.211	0.163
Southern Inland	0.203	0.204	0.204	0.203	0.186
Stanislaus	0.205	0.203	0.205	0.208	0.178
Tulare	0.200	0.206	0.202	0.200	0.192
Ventura	0.200	0.203	0.201	0.193	0.202

Appendix Table 3. Region-level forecasted incident cases and provisionally reported cases (as of December 2024) for 2023 and 2024. PI = prediction interval.

Region	Provisional Cases (as of Dec. 2024)	2023 Predicted (90% PI)	Provisional Cases (as of Dec. 2024)	2024 Forecasted (90% PI)
Bay Area	462	512 (469 – 568)	603	536 (494 – 594)
Central Coast	704	1,161 (999 – 1,385)	1,284	1,053 (944 – 1,195)
Eastern California	36	43 (31 – 73)	39	47 (32 – 80)
Northern California	25	34 (25 – 45)	58	24 (17 – 33)
Northern San Joaquin Valley	556	555 (472 – 644)	978	769 (649 – 883)
Southern Coast	2,174	2,968 (2,801 – 3,158)	2,458	3,076 (2,928 – 3,261)
Southern Inland	566	674 (611 – 745)	648	692 (628 – 761)
Southern San Joaquin Valley	4,273	5,331 (4,920 – 5,880)	6,383	5,162 (4,768 – 5,705)
Southern Sacramento Valley	81	148 (122 – 538)	139	151 (127 – 256)
**Statewide**	**9,212**	**11,426 (10,804–12,036)**	**12,590**	**11,509 (10,902–12,182)**

Appendix Table 4. Sensitivity analyses of our model specification.

Sensitivity analysis	R^2 compared to original model predictions	Mean absolute error compared to original model predictions
Removal of highly co-linear temperature variables (>12-mo lags)	0.98	0.034
Including a natural spline on year (df = 3) rather than as a linear term	0.90	0.012

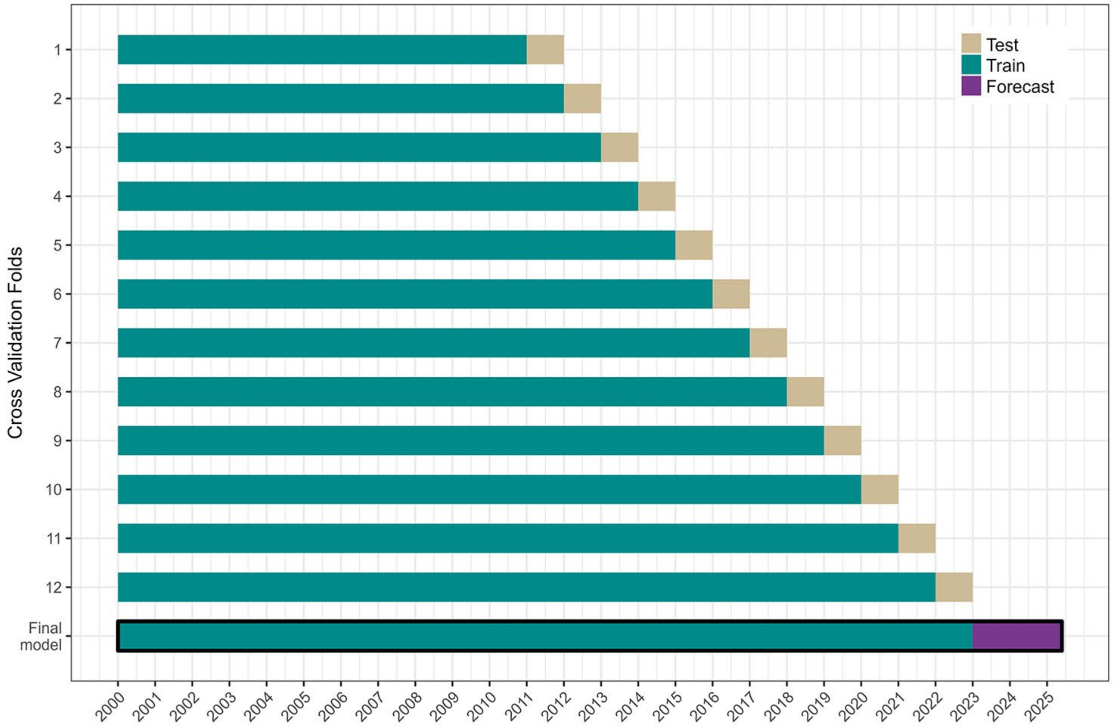

Appendix Figure 1. Schematic of the progressive time-series cross-validation approach used to generate weights for the ensemble model. Each progressive fold added an additional year to the training data (green) and predicted the following test year (beige). For example, we fit the models to data from 2000–2011 and predicted cases in 2012. We then fit the model to data from 2000–2012 and predicted cases in 2013, and so on. Individual models were then weighted by the inverse of their out-of-sample error across test years and used to forecast cases from Jan. 1st, 2023 – Mar. 31st, 2025 (purple).

A

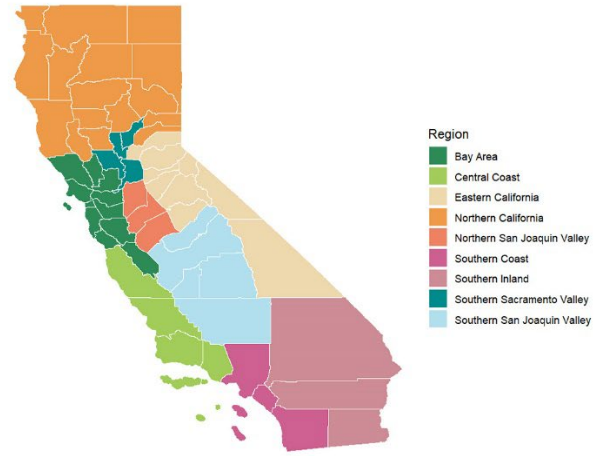

B

	Region	Counties
1	Bay Area	Alameda, Contra Costa, Napa, San Francisco, San Mateo, Santa Clara, Santa Cruz, Solano, Sonoma, Marin, San Benito
2	Central Coast	Monterey, San Luis Obispo, Santa Barbara, Ventura
3	Eastern California	Inyo, Mono, Mariposa, Tuolumne, Alpine, Calaveras, Amador, El Dorado, Placer
4	Northern California	Butte, Colusa, Del Norte, Glenn, Humboldt, Lake, Lassen, Mendocino, Modoc, Nevada, Plumas, Shasta, Sierra, Siskiyou, Tehama, Trinity
5	Northern San Joaquin Valley	Merced, San Joaquin, Stanislaus
6	Southern Coast	Los Angeles, Orange, San Diego
7	Southern Inland	Riverside, Imperial, San Bernardino
8	Southern Sacramento Valley	Sacramento, Yolo, Yuba, Sutter
9	Southern San Joaquin Valley	Fresno, Kern ,Kings, Madera, Tulare

Appendix Figure 2. Map of California counties by region. Starred regions are considered “high incidence”; counties within these regions were modeled independently.

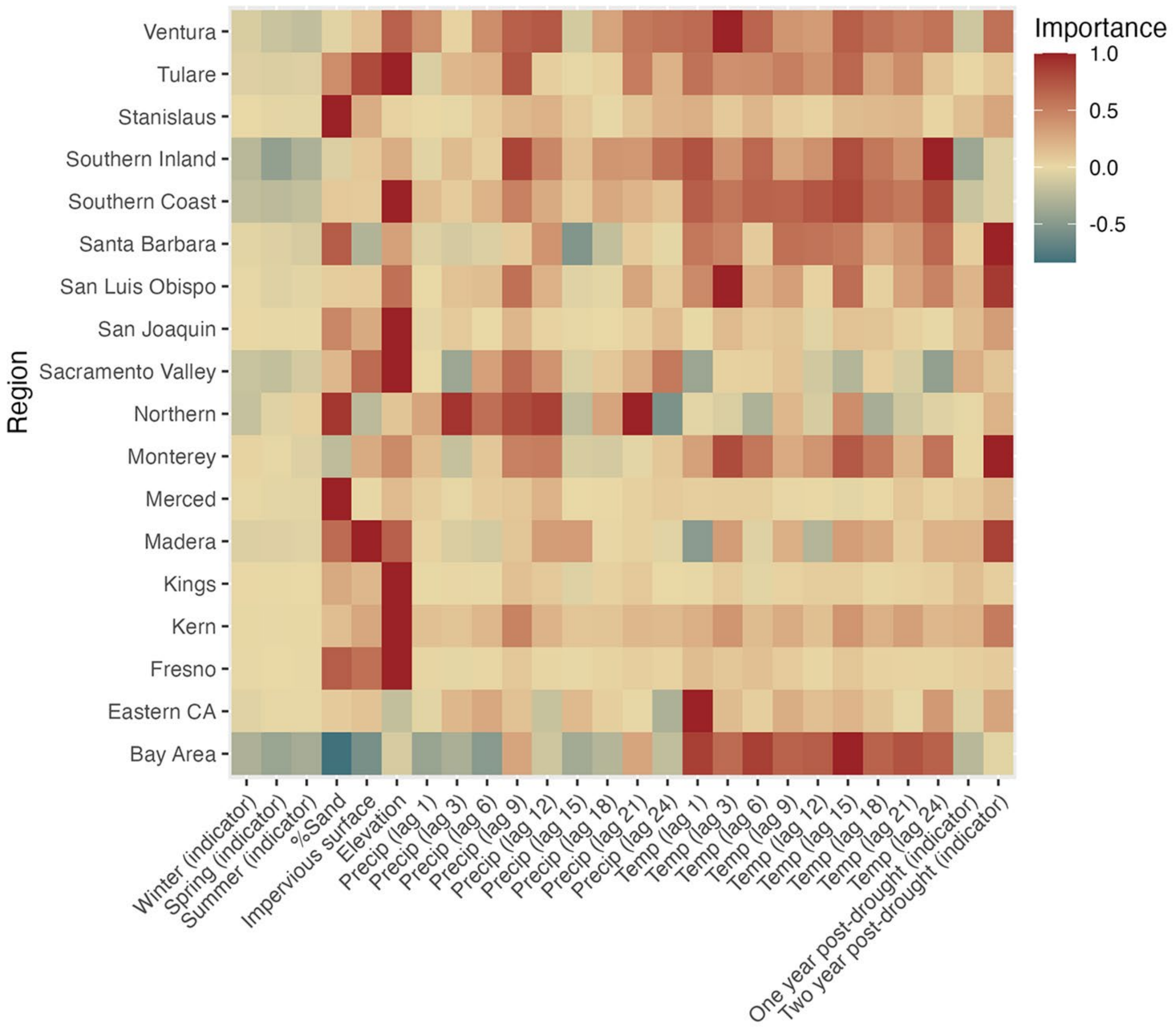

Appendix Figure 3. Corrected Gini Importance Indices from each region's random forest model. Index values have been scaled by dividing each region's index values by the regional maximum for comparability across regions.

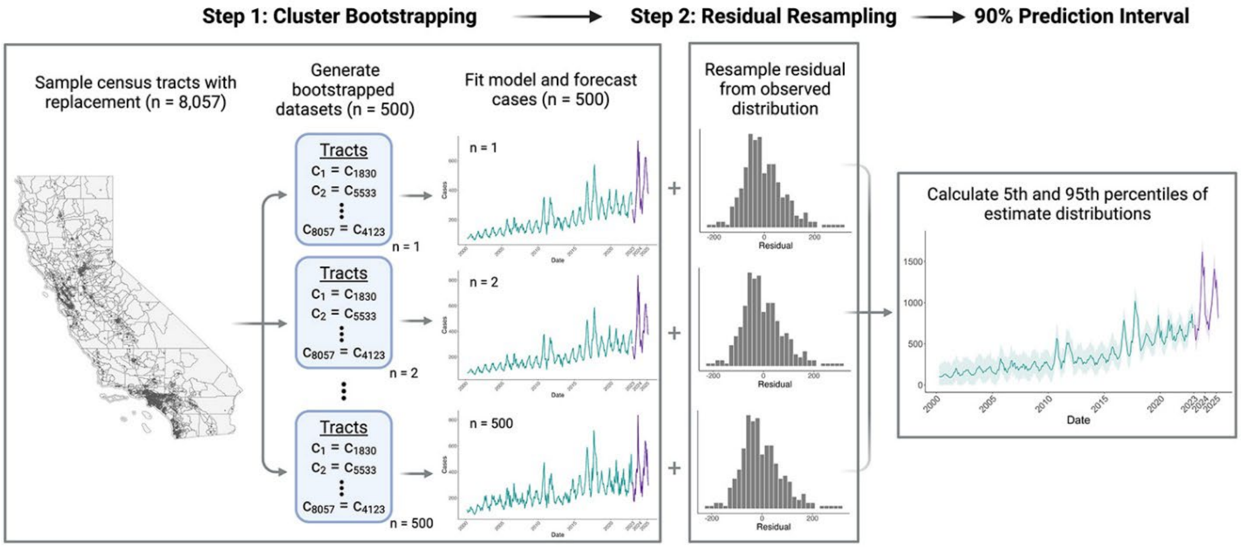

Appendix Figure 4. To generate 90% prediction intervals, we used a two-step bootstrapping process. In step 1, we sampled census tracts (n = 8057) with replacement to generate 500 datasets, fit the models to each dataset (n = 1–500), and forecasted cases using each model. In step 2, we then combined the estimates with resampled residuals from the observed residual distribution and calculated the 5th and 95th percentile of the resulting distribution to obtain 90% prediction intervals. Figure created with BioRender (https://www.biorender.com).
